# Textbook of Diseases of Infancy and Childhood

**Published:** 1910-12

**Authors:** 


**Textbook of Diseases of Infancy and Childhood.** By **Louis Fischer,** M.D., Attending Physician to the Willard Parker and Riverside Hospitals of New York. Third edition, Octavo, pp. 1003. With 333 illustrations and 29 full page plates. Philadelphia: F. A. Davis Co. 1910. (Cloth $6.50; half morocco, $8.00, net prices.)

The rapid exhaustion of the second edition stamps this volume as possessing in a high degree the essentials required in a work of this kind. To many the diagnosis and treatment of diseases of children is most perplexing, and the physician needs such a description of conditions as he will actually encounter at the bedside. Fischer has laid especial stress on the diagnostic symptoms that are so necessary, and of such assistance to the busy practitioner. Pathology and bacteriology have been given ample consideration. The new material in this revision is a more detailed consideration of the treatment of cerebrospinal meningitis with Flexner's antimeningetic serum. Intraspinal and intraventricular methods of treatment are illustrated and elaborated.

In septic diphtheria the intravenous injection of antitoxin has been added. The hemostatic value of horse serum injections in postoperative tonsillotomy is described. A new method for the preservation of human milk is given. Articles on scabies, indicanuria, pyuria, and diabetes necessitate the condensation of some of the less important subjects that appeared in former editions. It would seem that the four-page unrevised space allowed to infantile paralysis is rather brief in view of the great interest and enormous amount of literature on that subject at the present time. J. A. R.

